# Investigation of treatment efficacy of 10% povidone–iodine sclerotherapy on ovarian cyst diameter: an experimental study

**DOI:** 10.3906/sag-1810-27

**Published:** 2019-06-18

**Authors:** Remzi ATILGAN, Şehmus PALA, Tuncay KULOĞLU

**Affiliations:** 1 Department of Obstetrics and Gynecology, School of Medicine, Fırat University, Elazığ Turkey; 2 Department of Histology and Embryology, School of Medicine, Fırat University, Elazığ Turkey

**Keywords:** Povidone–iodine, cysts, ovarian reserve, rat

## Abstract

**Background/aim:**

The purpose of this study was to investigate the effects of 10% povidone–iodine (PI) sclerotherapy on ovarian cyst diameter through an experimental study.

**Materials and methods:**

To be able to create ovarian cysts, right unilateral salpingectomy was performed on 20 Wistar albino rats. One month after the operation, the abdomens of all rats were reopened. Sixteen rats with macroscopic ovarian cysts were divided randomly into 2 groups consisting of 8 rats. Group 1 (G1): the cyst content was only aspirated. Group 2 (G2): the ovarian cyst was aspirated and then the cystic cavity was irrigated with PI. Abdomens of all rats were closed and 1 month later they were reopened. Tissues of the right ovaries of the rats were embedded in paraffin blocks for histopathological examination. Follicle count, fibrosis, and congestion were evaluated under a light microscope.

**Results:**

For G1, there was no difference in cyst diameters before and after aspiration. In G2, a decrease was observed in cyst diameter. There was no difference in ovarian reserve between the 2 groups. When compared with G1, an increase in fibrosis and congestion was determined in G2.

**Conclusions:**

Sclerotherapy into the ovarian cyst for a 5-min period using 10% PI reduces cyst diameter without any change in ovarian reserve.

## 1. Introduction

Ovarian cysts are common, affecting 20% of women at some point in their lives (1). Unlike unilocular cysts including septations, solid irregular wall, or internal plaques, the simple ovarian cyst is defined as an anechoic round or oval lesion (2). The maximum diameter of simple ovarian cysts in premenopausal women is less than 5 cm; they often disappear during the menstrual cycle and do not require further intervention. Larger cysts (5–7 cm) should be followed using ultrasonography. Cysts larger than 7 cm may require advanced imaging or surgery (3). In the treatment protocols for benign ovarian cysts, methods such as medical treatment (especially oral contraceptives), ultrasound-guided aspiration, laparoscopy (cystectomy or ablation of drainage and cystic wall), and laparotomy (cystectomy) are used (4,5). 

Sclerotherapy can be used for women who have ovarian cysts but want to protect their fertility and do not want surgical treatment (6). It has been reported that sclerotherapy has many advantages such as minimal trauma, lower surgical risks and complications, low cost, and reduced recurrence rates (7,8). USG-guided aspiration and sclerotherapy have been reported as a low-cost and effective treatment for benign cysts localized in other organs such as the thyroid, parathyroid, liver, kidney, and spleen (9).

Tetracycline, methotrexate, and ethanol are the most common agents used for sclerotherapy. When compared to women without cysts, sclerotherapy applied to infertile women with ovarian cysts has been shown to reduce pelvic pain without affecting the number of follicles, term pregnancy and abortion rates, the number of obtained oocytes, embryo quality, or hormonal levels (10–13). 

Compared to other sclerosing agents, povidone–iodine (PI) is a less toxic, less irritating, economical, and readily available agent. In addition to local antiseptic, antibacterial, and antifungal effects, it has a sclerotherapeutic effect, and preparation of it at the desired concentration is easy (14). A 10% PI solution contains 1% available iodine, but the free iodine is at 0.1% concentration (15). In a systematic review, it was reported that iodine had no harmful effect on thyroid function and had no important side effects in terms of allergic reactions with iodine or cytotoxicity (16). However, in one study, it was shown that PI had a direct cytotoxic effect and stimulated necrosis in tissue (17). Sclerotherapy with PI is suggested as an effective, safe, and reproducible treatment method for symptomatic renal cysts (18) and peritoneal inclusion cysts (19). 

In this experimental study, it was aimed to investigate the effect of 10% PI instillation for 5 min on cyst diameter and ovarian reserve in ovarian cysts by taking advantage of its cytotoxic and tissue necrosis effects. 

## 2. Materials and methods

**Figure 1:**
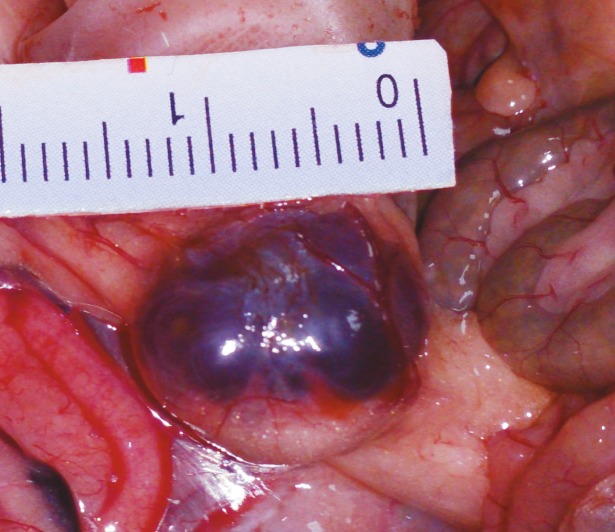
Ovarian cyst before aspiration.

**Figure 2:**
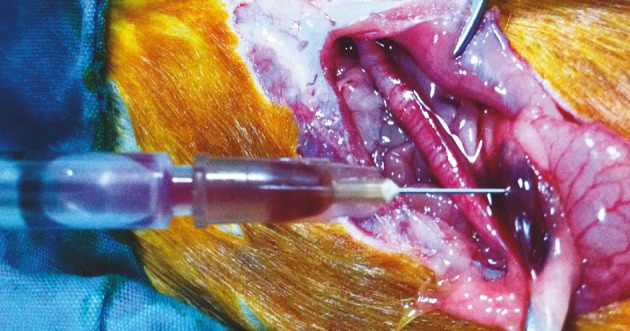
Ovarian cyst aspiration by insulin syringe.

This study was approved by Fırat University Animal Use Committee and conducted at Fırat University Animal Laboratory (Date: 2017/04; number of ethic committee approval: 50). Twenty mature, 12–14-week-old female Wistar albino rats having regular cycles and weighing 190–220 g were used in this study. Rats were kept under light and dark cycles for 12 h (from 07:00 to 19:00) at a room temperature of 21–23 °C in groups of 5 per cage as compatible to their biological rhythms. Standard pellets and tap water were used for feeding and watering the animals. Before the surgical interventions, oral feeding except water intake was stopped. Rats at the estrous stage of the cycle as documented by vaginal cytology were anesthetized using 70 mg/kg intramuscular ketamine (Ketalar, Eczacıbası, Turkey) and 10 mg/kg xylazine (Rompun, Bayer, Turkey). Surgical site antisepsis was achieved by using 10% PI. After midline laparotomy, right total salpingectomy was performed for induction of ovarian cysts as described by Atilgan et al. (20), and then the abdomen was closed with 3/0 silk sutures. One month after the first surgery, a second laparotomy was performed and macroscopic ovarian cysts were observed in 16 rats (80%) (Figure 1). Four rats in which macroscopic cysts were not observed were excluded from the study. The remaining 16 rats with macroscopic ovarian cysts were randomly divided into 2 groups: 

Group 1 (n = 8): the ovarian cyst was aspirated with an insulin injector and then the abdomen was closed (Figure 2).

Group 2 (n = 8): the ovarian cyst was aspirated with an insulin injector (Figure 2) and then the cystic cavity was irrigated with 10% PI at half of the aspirated cyst volume. Five minutes later, the 10% PI was reaspirated and the abdomen was closed. 

One month after the second laparotomy, 16 rats were decapitated just after the administration of intraperitoneal ketamine (75 mg/kg) + xylazine (10 mg/kg) and the laparotomy was performed. The presence and diameter of the ovarian cysts were recorded again for each rat, and right oophorectomy was then carried out for histological evaluations. Ovarian tissues were fixed in 10% formaldehyde and embedded in paraffin blocks; 4–6 µm sections were obtained from these blocks and stained with Masson’s trichrome to be able to determine ovarian follicle reserve, fibrosis, and congestion. Olympus BX50 light microscopy (Olympus Corporation, Tokyo, Japan) was used to determine and photograph the sections. Fibrosis score was determined as follows: 0 = none, +1 = mild, +2 = moderate, +3 = intense. The congestion in ovarian tissue was scored on a scale ranging from 0 to 3 (0: none; 1: mild; 2: moderate; 3: severe) (20). For follicular development, the method of Mazaud et al. (21) was used in microscopic classification. In the light microscopic examination of ovarian sections belonging to all groups, follicle classification was performed according to the following characteristics. Primordial follicle: oocyte is partially or completely surrounded by granulosa cells. Primary follicle: the follicle in which a single cubic granulosa cell layer is observed around the oocyte follicle. Antral (secondary) follicle: the follicle where the oocyte is covered with more than 2 layers of granulosa cells and where the formation of the antrum begins. Tertiary (Graafian) follicle: the follicle possesses a single large space (antrum) in which a decreasing number of granulosa cells surround an antrum full of follicular fluid, and that oocyte is surrounded by some granulosa cells (cumulus cells). 

### 2.1. Statistical analysis

For statistical analysis, SPSS version 22.0 (IBM Corporation, Armonk, NY, USA) software was used. Comparison of groups was carried out by using the Mann–Whitney U test, and the repetitive comparisons were done with the Wilcoxon rank test. P < 0.05 was considered statistically significant. 

## 3. Results

### 3.1. Macroscopic findings

**Figure 3:**
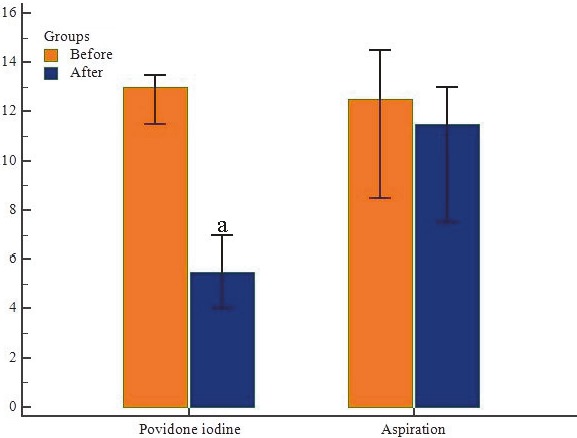
Cyst diameters before and after 10% PI administration (mm). Values are given as mean ± standard deviation. a = When compared with before 10% PI administration (P < 0.05).

When ovarian cyst size was evaluated in G1, there were no significant differences in terms of cyst diameters before (12.5 [7–14] mm) and after the aspiration (13 [9–16] mm) (P = 0.206). In G2, there was a statistically significant difference in cyst diameters before (11.5 [9–16] mm) and after the 10% PI administration (5.5 [0–15] mm), (P = 0.017) (Figure 3, Table 1). 

**Table 1:** Cyst diameters in both groups before and after application.

Rat’s number	Cyst diameters before aspiration (mm)	Cyst diameters after aspiration (mm)	Cyst diameters before PI administration (mm)	Cyst diameters after PI administration (mm)
1	14	12	13	8
2	15	13	16	5
3	13	14	11	3
4	11	10	12	15
5	19	13	13	6
6	6	5	9	5
7	5	3	13	0
8	12	11	14	6
Mean values	11.8 ± 4.6	10.1 ± 4.0	12.6 ± 2.0	6.0 ± 4.3a

Values are given as mean ± standard deviation. a When compared to before PI instillation group (P < 0.05).

### 3.2. Histological findings

The results of the examination of the ovarian tissue, which was stained with Masson’s trichrome, under the light microscope were as follows.

### 3.3. Congestion

**Figure 4:**
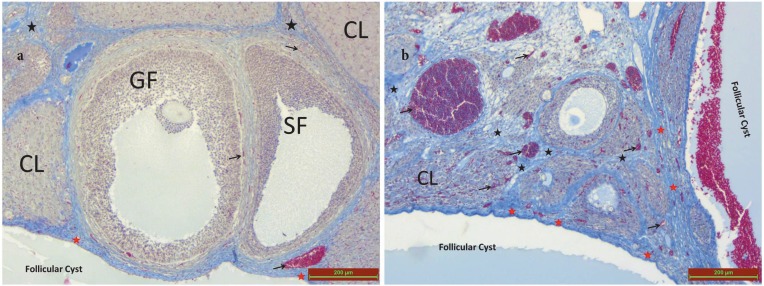
CL: corpus luteum, GF: graft follicle, SF: secondary follicle. Black arrow: congestion, red star: fibrosis near the cyst, black star: shows fibrosis in the tissue far away from the cyst.

When G1 (Figure 4a) was compared with G2 (Figure 4b), there was a statistically significant increase in congestion (black arrow) in G2 (P < 0.05) (Table 2).

**Table 2:** Histological score for fibrosis and congestion histoscore in both groups.

Groups	Fibrosis	Congestion
After aspiration	1.25 ± 1.03	0.87 ± 0.83
After 10% PI administration	2.62 ± 0.51a	2.25 ± 0.8a

Values are given as mean ± standard deviation. a When compared to the postaspiration group (P < 0.05).

### 3.4. Fibrosis

When G1 (Figure 4a) was compared with G2 (Figure 4b), there was a statistically significant increase in fibrosis in the cyst-adjacent area (red star) and parenchyma (black star) in G2 (P < 0.05) (Table 2).

### 3.5. Ovarian reserve

Comparing G1 and G2, there was no significant difference between the 2 groups in terms of the number of primordial (10.25 ± 2.31 / 9.25 ± 1.76), primary (9.37 ± 3.24 / 8.62 ± 2.61), secondary (4.25 ± 1.03 / 3.87 ± 0.64), and tertiary follicles (0.50 ± 0.53 / 0.37 ± 0.51), and the number of corpora lutea (11.50 ± 1.60 / 10.87 ± 2.10) (P > 0.05) (Figures 4a and 4b, Table 3).

**Table 3:** Number of follicles count in both groups.

Groups	Primordialfollicle	Primaryfollicle	Secondaryfollicle	Tertiaryfollicle	Corpusluteum
After aspiration	10.25 ± 2.31	9.37 ± 3.24	4.25 ± 1.03	0.50 ± 0.53	11.50 ± 1.60
After 10% PI administration	9.25 ± 1.76	8.62 ± 2.61	3.87 ± 0.64	0.37 ± 0.51	10.87 ± 2.10

Values are given as mean ± standard deviation. When compared to the postaspiration group (P > 0.05).

## 4. Discussion

The management of benign ovarian cysts is important in protecting fertility during the reproductive period. High recurrence rates in aspiration treatments have led researchers to treat these cysts by applying various sclerosing agents. In our study, we showed that 10% PI applied to simple ovarian cysts for 5 min reduced the cyst size significantly according to the aspiration-only application by increasing the congestion in the ovarian tissue and the fibrosis in the parenchyma tissue adjacent to the cyst. During the literature review in PubMed, no study on the administration of PI to ovarian cysts was identified. Therefore, it can be said that this study is original. 

Transvaginal aspiration of ovarian cysts has many advantages, such as excellent tolerability, low risk and cost, and low complication and recurrence rates. Therefore, it has been reported that cyst aspiration may be a reliable alternative to surgery (8,22–24). However, when the studies in the literature are examined, it is observed that aspiration-only models have higher recurrence rates than aspiration models using a sclerotherapeutic agent. Koutlaki et al. (22) reported that the transvaginal aspiration-only operation on simple ovarian cysts larger than 4 cm had a recurrence rate between 48% and 59%, depending on the patient groups. In another study, it was emphasized that the aspiration of simple ovarian cysts with transvaginal needle was safe. In the follow-up carried out for 1–6 months, the recurrence rate was reported as 39% in ovarian cysts. In the same study, the recurrence rate was reported as 62% in endometriomas, 35% in serous cysts, and 15% in serous–hemorrhagic cysts (8). It has been reported that sclerotherapy of simple ovarian cysts with ethanol without anesthesia in the office environment is an applicable technique with low complication and recurrence rates (23). At the end of a 12-month follow-up after the operation, Kars et al. (24) showed that transvaginal aspiration-only applied to nonneoplastic ovarian cysts had a recurrence rate of 50% and tetracycline sclerotherapy applied together with transvaginal aspiration had a recurrence rate of 14%. As in the literature, we did not find any significant reduction in cyst diameter in Group 1 at the end of this experimental study. 

It has been indicated that ethanol sclerotherapy, one of the medical treatment methods for cysts, has some complications such as intestinal perforation, intestinal stricture, neoplastic cells spreading to the peritoneal cavity, extensive pelvic adhesions, and pelvic bacterial infections (25). Due to the possible complications of the ethanol in cyst sclerotherapy, the efficacy of PI in sclerotherapy of ovarian cysts, which has antibacterial effects and is well-tolerated but not toxic or irritant, was investigated in this study (26).

It was shown that PI had success as a sclerosing agent in spaces with surrounded inline epithelium in recurrent fluid accumulation cases (26). It is reported that PI is a safe and effective sclerosing agent in lymphocele, pleural effusion, empyema, pericardial effusion, thyroid cysts, hydrocele, and lung Echinococcosis (26–28). PI was first used as a sclerotherapy agent by Teruel et al. (29). In that study, PI was instilled into the lymphocele via percutaneous catheters and the researchers found significant decreases in lymphocele diameters. Damianao et al. (30) aspirated about 300 ± 50 mL of lymphocele fluid with double-lumen catheters from 6 patients in whom lymphoceles developed after renal transplantation; they instilled 10 mL 5% PI in the recurrent periods. After the treatment, they reported a 100% success rate without any recurrences; they did not report any serious complications or infections. We thought that it would be very difficult to apply repetitive doses of PI or to place a percutaneous catheter in an experimental cyst model of rats. In their experimental sclerotherapy studies, Simsek et al. (31) applied a 95% ethanol instillation equal to half of the amount of cyst fluid that they aspirated to simple ovarian cysts for 5 min. Similarly, we determined the 10% PI instillation time as 5 min.

Lim et al. (19) carried out sclerotherapy in 30 patients with peritoneal inclusion cysts. After cyst aspiration, they applied 10% PI as a sclerotherapy agent in 13 patients, and they applied pure ethanol in 17 patients for sclerotherapy. The maximum amount of sclerosing agent given was 100 mL and did not exceed one-third of the cyst fluid they aspirated. In the long term, there was a 90% success rate and secondary infections developed in only 2 patients who did not use prophylactic antibiotics. At the end of the study, it was reported that both PI and ethanol, as sclerosing agents used for sclerotherapy, had similar successful effects on cyst diameter. 

Madeb et al. (32) suggested that treatment of symptomatic simple renal cysts with PI sclerotherapy was not indicated because of high recurrence rates. In contrast, Harrach et al. (18) showed that sclerotherapy with PI had high success rates in treatment of simple renal cysts. In our study, while a significant decrease in ovarian cyst diameter with 10% PI sclerotherapy was observed when compared with the control (aspiration) group, no complications such as macroscopic abdominal organ damage or abdominal abscess formation were identified. In this study, in histopathological examination of ovarian tissue after instillation of 10% PI as the sclerosing agent, a significant increase in fibrosis which was more prominent in areas neighboring the cyst wall was found when compared with the aspiration-only group. We think that this histopathological effect is a result of PI damaging the cyst wall and neighboring ovarian tissue. The increased congestion in neighboring ovarian tissue supports our findings. In the previous studies mentioned above, the effects of PI sclerotherapy were investigated; however, since they did not evaluate the histopathological effects of PI sclerotherapy in cyst-adjacent areas, they did not give any information about possible damage to neighboring tissues. 

The term ovarian reserve is defined as the number and quality of follicles remaining in the ovaries at any given time (33). In a prospective randomized study, it was shown that cyst wall ablation may be a more appropriate method than cystectomy in terms of preservation of ovarian function (34). Recently, it has been shown that the application of ethanol sclerotherapy after ultrasound-guided aspiration reduces recurrence rates and does not adversely affect reproductive outcomes (10,35,36). Although Atilgan et al. (20) reported a significant decrease in ovarian cyst diameters with 10 min of 10% ethanol sclerotherapy instillation in an experimental rat model, they also found a significant decrease in primordial, primary follicle count and ovarian reserve in the ethanol-instilled group when compared with the aspiration-only group. The same study showed that 10% ethanol application significantly increased fibrosis in ovarian tissue. Simsek et al. (31) also showed that 95% ethanol sclerotherapy applied for a 5-min period reduced the size of ovarian cysts significantly; however, it did not lead to a significant difference in the total follicle number or fibrosis. With these two studies, it is understood that the instillation time of a sclerotherapeutic agent such as ethanol may be associated with increased ovarian damage. As a result of the ovarian cyst sclerotherapy conducted using trichloroacetic acid (TCA) by Artas et al. (37), it was reported that a 5-min 5% TCA application led to a significant decrease in the size of the cysts without causing any significant decrease in ovarian reserve. They also showed that in the group to which 5% TCA was applied, there was a significant increase in fibrosis and congestion in the adjacent ovarian tissue and parenchymal. In our study, instillation of 5 min of 10% PI sclerotherapy resulted in an increase in congestion and fibrosis in ovarian tissue, but any decrease in ovarian reserve was not observed. Souza et al. (38) reported that the increase in fibrosis could be a compensatory mechanism which prevents the reduction in ovarian reserve. This may explain the similar follicle numbers determined in the groups despite the differences in congestion and fibrosis. Thus, these results show that ethanol sclerotherapy as administered by Atilgan et al. (20) was more destructive than PI sclerotherapy, and PI can be used more safely in simple ovarian cysts. 

In an experimental pulmonary aspiration study, the instillation of lung tissue with PI was shown to cause atelectasis depending on the dose. Based on the primary light and scanning electron microscopic findings in the study, the scholars showed that edema had formed in the pulmonary interstitium and that scar tissue formation and alveolar rupture had occurred as a result of leukocyte infiltration (39). In our study, congestion in ovarian stroma and an increase in fibrosis was found in the group in which PI was used. However, it was also seen that these effects did not result in a significant decrease in the number of follicles. This may be because the cyst wall, by acting like a barrier, prevents the destructive effect of PI from reaching distant tissue areas. 

The fact that 10% PI sclerotherapy was applied to the ovarian cysts for the first time in our study and that ovarian histopathological changes were examined are strengths of our study. On the other hand, our study was an experimental study, the results obtained in animal models cannot replicate human results due to the differences between species, and the number of cases was limited, which are limitations of the study.

In conclusion, sclerotherapy with 10% PI applied into the ovarian cyst for 5 min significantly reduces cyst diameter without a significant alteration in ovarian reserve. Additionally, 10% PI is safe and well tolerated.
